# Success of *Helicobacter pylori* Guideline-based Treatment of Newly Diagnosed and Previously Treated Patients During 2007–2021 in Edmonton, Alberta

**DOI:** 10.1093/jcag/gwad051

**Published:** 2023-12-14

**Authors:** Thomas Krahn, Jonas Buttenschoen, Pernilla D’Souza, Safwat Girgis, Aducio Thiesen, Robert Rennie, LeeAnn Turnbull, Sander Veldhuyzen van Zanten

**Affiliations:** Division of Gastroenterology, Department of Medicine, University of Alberta, Edmonton, Canada; University of Erlangen-Nuremberg, Erlangen, Germany; Division of Gastroenterology, Department of Medicine, Trillium Health Partners, Mississauga, Canada; Department of Laboratory Medicine and Pathology, University of Alberta, Edmonton, Canada; Department of Laboratory Medicine and Pathology, University of Alberta, Edmonton, Canada; Department of Laboratory Medicine and Pathology, University of Alberta, Edmonton, Canada; Alberta Precision Laboratories, Edmonton, Canada; Division of Gastroenterology, Department of Medicine, University of Alberta, Edmonton, Canada

**Keywords:** Helicobacter pylori, antibiotic resistance

## Abstract

**Background:**

Updated 2016 *Helicobacter pylori* consensus guidelines recommend treatment for 14 days with concomitant therapy (proton-pump inhibitor (PPI)-amoxicillin-metronidazole-clarithromycin (PAMC) or bismuth-based quadruple therapy (PPI-bismuth-metronidazole-tetracycline, PBMT)) as first line, PBMT or PPI-amoxicillin-levofloxacin (PAL) as second or third line, and PPI-amoxicillin-rifabutin (PAR) as fourth line for 10 days.

**Objectives:**

This was a retrospective cohort study to describe and compare the efficacy of anti-*Helicobacter* treatment regimens over the periods 2007–2015 and 2016–2021 as well as antibiotic resistance.

**Methods:**

A modified intention-to-treat (mITT) analysis was used to analyze the success rate of therapies. mITT includes all patients who were prescribed *H. pylori* treatment and had at least one follow-up test-of-cure. This included patients who could not complete treatment or were non-adherent with treatment. Risk factors for treatment failures were analyzed by univariate and multivariate logistic regression. Resistance testing was done in a small subset of patients.

**Results:**

*H. pylori*-positive patients who received treatment in Edmonton, Alberta were included in a mITT analysis: 334/387(86%) from 2007 to 2015 and 193/199 (97%) from 2016 to 2021. During 2016–2021, 78% (150/193) of patients underwent cumulative guideline-based treatment with a successful cure in 80% (120/150) of patients. In those who were newly diagnosed, the cure rate was 88% (52/59) versus those with previous treatment failure 75% (68/91) (*P* < 0.05, risk difference [RD] 14%, 95% confidence interval [CI] 1.7–26.3%). The most effective first-line regimens were PAMC for 14 days (87% [45/52]) in 2016–2021 and sequential therapy in 2007–2015 (83% [66/80]) (*P* = 0.535, RD 4%, 95% CI −8.5–16.5%). When other treatments failed, success with PAR was 50% (2/4) from 2007 to 2015 and 57% (21/37) from 2016 to 2021. Recent (2016–2021) resistance rates to clarithromycin and metronidazole are high at 78% (50/64) and 56% (29/52), respectively. From 2007 to 2015, clarithromycin and metronidazole resistance rates were 80% (36/45) and 83% (38/46), respectively. Levofloxacin resistance increased significantly from 2007–2015 to 2016–2021 (28% [13/46] to 61% [35/57], *P* < 0.05, RD 33%, 95% CI 11.6–54.4%).

**Conclusions:**

Algorithmic treatment with PAMC first line followed by PBMT, PAL, and PAR cures *H. pylori* in 88% of newly diagnosed patients. PAR therapy shows suboptimal cure rates (50–57% success) but can be considered as third instead of fourth line given increasing levofloxacin resistance rates. Antibiotic resistance in *H. pylori* is common to clarithromycin, metronidazole, and levofloxacin and frequently accounts for treatment failures.

## Introduction

*Helicobacter pylori* is an accepted cause of gastritis, dyspepsia, duodenal, and gastric ulcers and is a risk factor for gastric cancer. There is consensus that *H. pylori-*positive patients should be offered treatment.

Traditionally, therapy involved “classic” triple combinations of a proton pump inhibitor (P) with clarithromycin (C) and either amoxicillin (A) (PAC) or metronidazole (M) (PMC) for 7–14 days. These two combinations previously had efficacy rates >80–90%.^1,2^ However, these combination therapies are no longer recommended because of low success rates mainly attributed to resistance of *H. pylori* to antibiotics, particularly clarithromycin and also metronidazole.^3^

Consequently, new treatment combinations have evolved. The 2016 Toronto *H. pylori* consensus and other international guidelines all make similar updated recommendations,^4–7^ including treatment duration for 14 days. First-line therapies are concomitant therapy with a proton-pump inhibitor (PPI) and amoxicillin-metronidazole-clarithromycin (PAMC) or bismuth-based quadruple therapy (PPI-bismuth-metronidazole-tetracycline, PBMT). Recommended second and/or third-line therapies are PBMT or PPI-amoxicillin-levofloxacin (PAL) and the fourth-line PPI-amoxicillin-rifabutin (PAR) given for 10 days.

Few studies have reported on the efficacy of *H. pylori* regimens in Canada, levels of antibiotic resistance, and changes over time.

The objectives of the study were to (1) compare the efficacy of different anti-*Helicobacter* regimens over the periods 2007–2015 and 2016–2021, (2) report the success rates of regimens when given as first-, second-, or higher-order treatments, (3) determine the cumulative success rate when patients were treated in sequence with different regimens, (4) provide data on antibiotic resistance, and (5) determine predictors of treatment success.

## Methods

This retrospective study reviewed records of patients treated for *H. pylori* infection at the University of Alberta Hospital (UAH), a tertiary referral centre in Edmonton, Alberta from January 1, 2007 to June 1, 2021. Given recent updates to clinical practice guidelines, therapeutic regimens from a previous era of treatment (2007–2015) are compared to the current era of treatment (2016–2021). Separately, data on antibiotic resistance in *H. pylori* are reported from 1999 to 2021. The Strengthening the Reporting of Observation Studies in Epidemiology (STROBE) guidelines were used in the reporting of data.^8^

### Patients

Patients seen at UAH were either newly diagnosed or previously treated for *H. pylori* infection. Participants were eligible for study inclusion if they were ≥18 years of age and had a diagnosis of *H. pylori* infection confirmed by at least one of a ^13^C urea breath test (UBT), histology, culture, or Helicobacter stool antigen test (HpSAT). Exclusion criteria included age < 18 years or if there was no documented test of the presence of *H. pylori* infection prior to treatment. One physician (SVvZ) treated the vast majority (90%) of patients, so no comparisons were made among physicians regarding treatment choices.

Data were collected on patient demographics, method of *H. pylori* diagnosis, treatment regimen(s) prescribed, duration of therapy, post-treatment eradication testing, number of courses of antibiotics prescribed for *H. pylori*, and success of therapy. For all patients, it was determined whether they had received anti-*Helicobacter* treatment before they were referred to UAH and what regimens were used, although it was not always possible to get precise information on previous treatment regimens. Compliance and side effects were not formally assessed. They were only recorded if patients reported problems in taking all the prescribed medications or if treatment was stopped because of side effects.

### Diagnostic tests used

Diagnosis of *H. pylori* infection and test-of-cure (TOC) were documented with a ^13^C UBT, HpSAT, or positive histology on endoscopic biopsies. For some patients, more than one test was available. For histology routine, hematoxylin and eosin staining was used, and sometimes, a modified Giemsa or Warthin-Starry (silver stain) was used at the discretion of the pathologist. A well-validated ^13^C UBT was used (Helikit^R^ UBT, Isodiagnostika, Edmonton, Canada). In all patients, TOC was performed at least > 4 weeks after the anti-*Helicobacter* therapy was completed. In the majority of patients, repeat testing was done 4–8 weeks after anti-*Helicobacter* therapy was completed.

Cultures of *H. pylori* were available in some patients but never used alone to establish diagnosis. Details on cultures of *Helicobacter pylori* and resistance testing are listed in the Supplementary File.

### Data analysis

**Figure 1.**
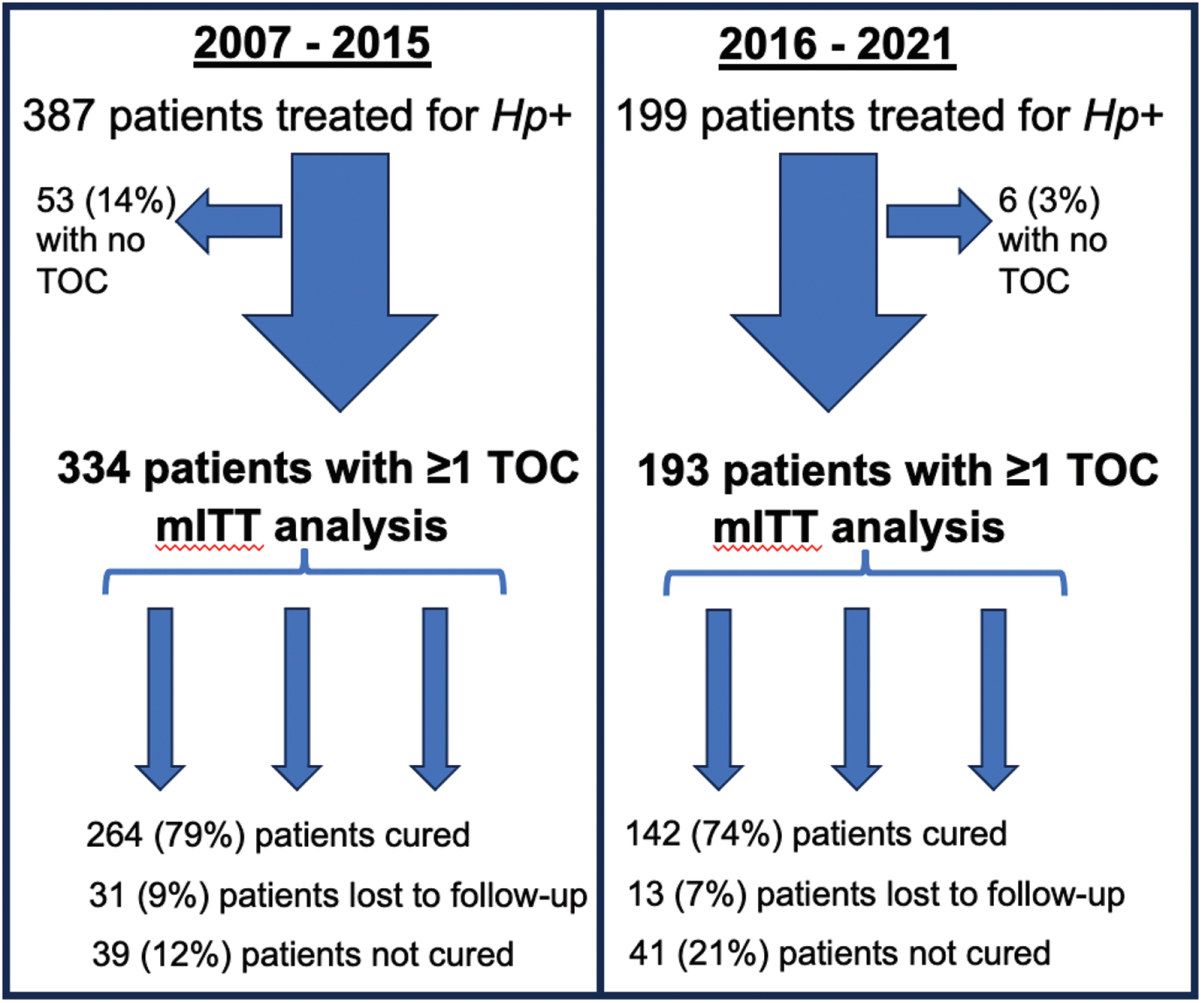
All included patients treated for *H. pylori* infection. *Hp, Helicobacter pylori*; TOC, test-of-cure; mITT, modified intention-to-treat.

For treatment results, a *modified intention-to-treat* (mITT) analysis was used that included all patients in whom treatment was prescribed with at least one subsequent TOC (Figure 1). This included patients who could not complete treatment or were non-adherent with treatment. The Chi-square test was used to determine *P*-values for categorical variables and the *T*-test used for numerical variables. For all treatment results, 95% confidence intervals (CI) were calculated.^9^ For risk factor analysis of treatment results, univariate and multivariate logistic regression was performed in SPSS (IBM®).

### Reporting of results

Cumulative results are reported separately for those patients who received their first guideline-based anti-*Helicobacter* treatment at UAH (newly diagnosed, treatment naive) and treated according to the guidelines, i.e., sequential therapy during 2007–2015 and concomitant therapy (PAMC) during 2016–2021 as first line. If these therapies failed, further treatments were also guideline-based. Separately, results are also reported for those patients who had received one or more treatments prior to being seen at UAH. Their subsequent choices of therapy were also guideline-based. For example, if a patient had failed a clarithromycin-based regimen, use of clarithromycin was avoided in subsequent treatments. In those patients in whom cultures and resistance data were available, the treatment choice was also guideline-based. If a patient’s *H. pylori* strain was resistant to clarithromycin or levofloxacin, those regimens were avoided. If a patient was resistant to metronidazole but sensitive to levofloxacin, PAL was given before PBMT. Resistance to amoxicillin was not used in treatment decisions.

The study was approved by the Research Ethics Review Board at the University of Alberta. As this was a retrospective study with a de-identified analysis of patient data, the need for individual patient consent was waived.

## Results

Table 1 lists the various regimens that were used including dosages. It should be noted that in 2016, duration was changed for most treatments from 10 to 14 days except for Rifabutin-based therapy (PAR). The difference between Sequential and Concomitant (PAMC) therapy was not only the duration of therapy (10 vs. 14 days) but also the duration of antibiotics given. For sequential therapy, amoxicillin is given for 5 days (day 1–5) followed by 5 days clarithromycin and metronidazole (day 6–10). In concomitant therapy, all three antibiotics are given for the full 14 days.

**Table 1.** Description of the treatment regimens used for *H. pylori* infection.

	Treatment	Medications	Dose used	Duration
Currently recommended **first** line therapy	Concomitant Therapy (PAMC)	1) Proton Pump Inhibitor 2) Amoxicillin 3) Clarithromycin 4) Metronidazole	1 tablet BID 1 g BID 500 mg BID 500 mg BID	14 days
Currently recommended **first** or **second** line therapy	Bismuth based Quadruple therapy (PBMT)	1) Proton Pump Inhibitor 2) Bismuth-subsalicylate 3) Metronidazole 4) Tetracycline	1 tablet BID 500 mg QID^a^ 500 mg QID 500 mg QID	14 days
	PPI-Amoxicillin-Levofloxacin (PAL)	1) Proton Pump Inhibitor 2) Amoxicillin 3) Levofloxacin	1 tablet BID 1 g BID 500mg BID^b^	14 days
Currently recommended rescue therapy	Rifabutin based triple therapy (PAR)	1) Proton Pump Inhibitor 2) Amoxicillin 3) Rifabutin	1 tablet BID 1 g BID 150 mg BID^c^	10 days
Previously recommended antibiotic regimens	Triple therapy (PAC)	1) Proton Pump Inhibitor 2) Amoxicillin 3) Clarithromycin	1 tablet BID 1 g BID 500 mg BID	7–14 days
	Triple therapy (PMC)	1) Proton Pump inhibitor 2) Metronidazole 3) Clarithromycin	1 tablet BID 500 mg BID 500 mg BID	7–14 days
	Sequential Therapy	1) Proton Pump Inhibitor 2) Amoxicillin days 1–5/7 3) Clarithromycin 6/8–10/14 4) Metronidazole days 6/8–10/14	1 tablet BID 1 g BID 500 mg BID 500 mg BID	10–14 days

Please see notes below for doses that differed from the recommended doses by the Toronto consensus guidelines ^4^

^a^Recommended dose of Bismuth subsalicylate is 250–500 mg PO QID.

^b^Recommended dose of Levofloxacin is 250 mg PO BID.

^c^Recommended dose of Rifabutin is 150 mg PO qd.

Figure 1 shows all patients screened for inclusion in this study, including exclusions.

Table 2 displays the demographics of the 199 patients who were assessed at UAH for *H pylori* between January 1, 2016 and June 1, 2021. Included subjects were predominantly female (67%, 134/199), and (50%, 99/199) were born outside of Canada, with a median age of 49 years old (interquartile range [IQR] 35.5–59). There was no TOC available for 3% of the subjects (6/199), so these were excluded from the analysis. Of the 193 included in the mITT analysis, 7% (13/193) were lost to follow-up, and 74% (142/193) were cured. In the 2007–2015 cohort, there were 387 patients treated initially; 57% (221/387) were female with a median age of 51 years (IQR 51–63). In this cohort, 86% (334/387) had at least one follow-up TOC, with an overall cure rate of 79% (264/334).

**Table 2.** Demographics of patients evaluated for *H. pylori* treatment (2016–2021).

	Subjects (*n* = 199)
Age (median)	49 (IQR 36–59)
Female sex	67% (134/199)
Born outside of Canada	50% (99/199)
Treated first at UAH	39% (77/199)
Median number of treatments received	3 (IQR 1–4)
**Diagnosis of *H. pylori* infection** (More than 1 test possible)	
Urea Breath Test	76% (151/198)
Endoscopy	47% (94/198)
Culture	11% (21/198)
Stool antigen test	0.5% (1/198)
**Allergies**	
Penicillin allergy	6% (12/199)
Tetracycline allergy	1.5% (3/199)
Clarithromycin allergy	0.5% (1/199)
Metronidazole intolerance	0.5% (1/199)
**Reason for referral** (More than 1 reason possible)	
Dyspepsia	25.1% (50/199)
Persistent *H. pylori*	57.3% (114/199)
New diagnosis *H. pylori*	9% (18/199)
GERD	7.5% (15/199)
Iron deficiency anemia	5.5% (11/199)
GI Bleeding/Ulcer disease	6.5% (13/199)
Nausea/vomiting	1.5% (3/199)
**PPI used**	
Pantoprazole	83% (164/197)
Omeprazole	10% (19/197)
Lansoprazole	5% (9/197)
Esomeprazole	2% (3/197)
Rabeprazole	1% (2/197)
**Known Antibiotic exposures**	
Clarithromycin	58% (115/199)
Metronidazole	40% (79/199)
Levofloxacin	21% (42/199)
Amoxicillin	55% (109/199)
Normal	56% (86/155)
Gastritis or duodenitis	26% (40/155)
Gastric or duodenal ulceration	10% (16/155)
Intestinal metaplasia on biopsy	7% (10/155)
Esophagitis	5% (8/155)
Gastric cancer	0.6% (1/155)
Lymphoma	0.6% (1/155)
Changes consistent with celiac disease	3% (5/155)

During 2016–2021, 78% (150/193) of patients underwent cumulative guideline-based treatment with a successful cure in 80% (120/150) of patients. In those who were treatment-naive cure rate was 88% (52/59) versus those with previous treatment failure, 75% (68/91) (*P* < 0.05, risk difference [RD] 14%, 95% CI 1.7–26.3%).

**Figure 2.**
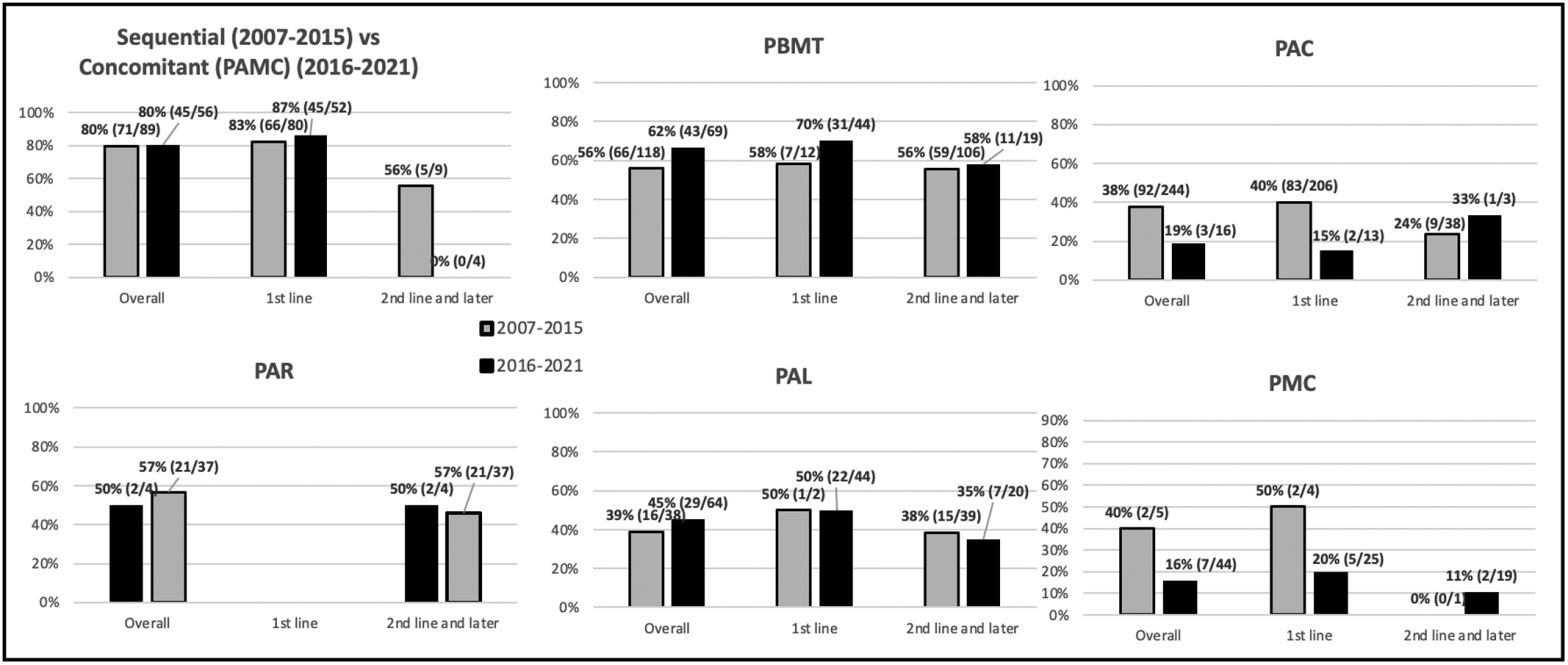
Comparative success rates of *H. pylori* treatment regimens during the 2007–2015 and 2016–2021 periods in newly diagnosed and previously treated patients (modified intent-to treat analysis, with all subjects included in analysis unless there was no follow-up/test-of-cure after their first treatment regimen).

Figure 2 shows a comparison of the success rates of different therapies for the two guideline periods of 2007–2015 and 2016–2021. The success rate of PAMC therapy as first line in 2016–2021 (87%, 45/52) was numerically superior to sequential therapy (83%, 66/80) in 2007–2015 (*P* = 0.535, RD 4%, 95% CI −8.5–16.5%). As first line, the success rate of PBMT was 58–70% (figure 2). The success rates of first line PAC and PMC were low at 40% and 50%.

Bismuth-based quadruple therapy, when given as second-line therapy, had an efficacy of 56% (59/106) during 2007–2015 and 58% (11/19) during 2016–2021. PAL as second line or later therapy had a cure rate of 38% (15/39) in 2007–2015 and 35% (7/20) in 2016–2021. Overall treatment success of second line or later PBMT was greater than PAL (56% [70/125] vs. 37% [22/59], *P* < 0.05, RD 19%, 95% CI 5.1–32.9%).

The success rates of the Rifabutin combination used after patients failed treatment with standard therapies were 50% (2/4) in 2007–2015 and 57% (21/37) in 2016–2021. Overall treatment success with PAL and PAR was not significantly different (*P* = 0.0783, RD 19%, 95% CI −0.34–38.3%), but the sample size for this comparison was small.

The data demonstrate that if guideline-based therapies are followed in sequence, high success rates can be achieved after three or four rounds of therapy. The cure rate of the cumulative guideline-based approach of patients first treated at UAH was 86% (69/80) during 2007–2015 (Sequential—PBMT—PAL—PAR) and 88% (46/52) during 2016–2021 (PAMC—PBMT—PAL—PAR).

Logistic regression was used to identify predictors of treatment success (Table 3). Univariate analysis identified being treated first at UAH as a predictor of treatment success (odds ratio [OR] = 3.177, *P* = 0.003, 95% CI 1.476–6.840). Previous exposure to clarithromycin (OR = 0.461, *P* = 0.03, 95% CI 0.299–0.927), metronidazole (OR = 0.232, *P* < 0.001, 95% CI 0.117–0.458), and amoxicillin (OR = 0.441, *P* = 0.19, 95% CI 0.222–0.876) were negatively associated with successful treatment. The forward selection (likelihood ratio) method was used to select being born outside Canada, and previous Metronidazole exposure as variables for multivariate analysis, with treated first at UAH added to the model due to the strength of effect on univariate analysis. Previous metronidazole exposure was negatively associated with treatment success (OR = 0.290, *P* = 0.007, 95% CI 0.118–0.713); however, other antibiotic exposures did not add to the model. Being treated first at UAH was not an independent predictor of cure on multivariate analysis (OR 1.687, *P* = 0.326, 95% CI 0.595–4.785). Overall, 75% (8/12) of penicillin-allergic patients were cured, with 5/6 (83%) of them cured with first-line PBMT.

**Table 3.** Predictors of treatment success in patients treated for *H. pylori* 2016–2021, logistic regression.

	Included subjects (*n* = 199)	Cured (*N* = 142)	Not cured (*N* = 51)	Univariate analysis			Multivariate analysis		
				OR	(95% CI)	*P*-value	OR	(95% CI)	*P*-value
Age (median)	49 (IQR 36–59)	48 (IQR 34–58)	53 (IQR 40–62)	0.982	(0.962–1.003)	0.093			
Female gender	134 (67%)	97 (68%)	33 (65%)	1.176	(0.599–2.308)	0.638			
Birth outside of Canada	99 (50%)	76 (54%)	20 (39%)	1.785	(0.930–3.425)	0.081	**2.257**	**(1.107–4.602)**	**0.025**
Treated first at UAH		62 (44%)	10 (20%)	**3.177**	**(1.476**–**6.840)**	**0.003**	1.687	(0.595–4.785)	0.326
Penicillin allergy	12 (6%)	8 (6%)	4 (8%)	0.701	(0.202–2.437)	0.577			
**Antibiotic exposures**									
Clarithromycin	110 (60%)	78 (55%)	37 (73%)	**0.461**	**(0.299**–**0.927)**	**0.030**			
Metronidazole	74 (40%)	45 (32%)	34 (67%)	**0.232**	**(0.117**–**0.458)**	**<0.001**	**0.290**	**(0.118**–**0.713)**	**0.007**
Levofloxacin	40 (22%)	28 (20%)	14 (28%)	0.659	(0.309–1.362)	0.253			
Amoxicillin	104 (59%)	73 (51%)	36 (71%)	**0.441**	**(0.222**–**0.876)**	**0.019**			

CI, confidence interval; OR, odds ratio.

The results of bacterial cultures and antibiotic resistance levels are reported in Table 4. Data from Nova Scotia from 1999 to 2006 is described that was previously only reported in abstract form^11^ with changes in primary resistance to clarithromycin from 1990 to 1997 published previously.^10^

**Table 4.** *H. pylori* antibiotic resistance rates in Canada from 1999 to 2021.

	1999–2006		2007–2015		2016–2021	
Treatment	Primary	Secondary	Primary	Secondary	Primary	Secondary
Amoxicillin	0 (0/157)	0 (0/157)	0 (0/5)	0 (0/41)	10 (1/10)	8 (3/36)
Clarithromycin	15 (37/245)	56 (137/245)	60 (3/5)	83 (33/40)	59 (10/17)	85 (40/47)
Metronidazole	25 (63/253)	52 (132/253)	40 (2/5)	88 (36/41)	63 (10/16)	53 (19/36)
Levofloxacin	19 (30/157)	5 (9/157)	20 (1/5)	29 (12/41)	69 (9/13)	59 (26/44)
Tetracycline	n/a	n/a	0 (0/5)	0 (0/41)	0 (0/16)	0 (0/47)

Data from 1999 to 2006 were collected from Halifax, Nova Scotia; data from 2007 to 2021 were collected from Edmonton, Alberta. For details, see text. Determination of resistance based on European Committee on Antimicrobial Susceptibility Testing (EUCAST) breakpoints for *H. pylori* antibiotic resistance. Reported cases of amoxicillin resistance were all slightly above the EUCAST MIC of 0.0125 mg/L.^10^.

Primary resistance to clarithromycin was 59% (10/17) from 2007 to 2015 and 60% (3/5) from 2016 to 2021 in Edmonton, Alberta. Metronidazole resistance was present in 83% (38/46) and 56% (29/52) of cultures obtained in 2007–2015 and 2016–2021, respectively. Of the cases resistant to clarithromycin, 79% (11/14) were successfully treated with PBMT compared to 0% (0/3) of cases sensitive to clarithromycin. Of the cases resistant to metronidazole, 50% (2/4) were successfully treated with PBMT, whereas 67% (4/6) of cases sensitive to metronidazole were successfully treated with PBMT. Supplementary Table 1 describes the details of the outcome of treatments of patients with dual resistance. There were 23/50 (46%) cultures of dual clarithromycin and metronidazole resistance. In patients with dual resistance who were further treated, 37% (7/19) were cured, and of those who received PBMT, 67% (2/3) were successfully treated. Levofloxacin resistance increased significantly from 2007–2015 to 2016–2021 (28% [13/46] to 61% [35/57], *P* < 0.05, RD 33%, 95% CI 11.6–54.4%).

No cases of resistance to tetracycline were observed between 2007 and 2021. Resistance to amoxicillin was not seen in any of the 157 strains analyzed in Halifax during 1999–2006^11^ or in the 41 Edmonton strains obtained during 2007–2015. There were four cases of amoxicillin resistance during 2016–2021: 10% (1/10) primary resistance and 8% (3/36) secondary resistance. All cases of amoxicillin resistance were slightly above the European Committee on Antimicrobial Susceptibility Testing (EUCAST) MIC of 0.0125 mg/L.^12^ It is unclear whether this signifies true amoxicillin resistance.

## DISCUSSION

Despite the fact that *H. pylori* is an important pathogen, relatively few data have been published over the last 15 years on the efficacy of anti-*Helicobacter* treatments in Canada.^7–9,11^ In 2016 and 2017, several updated treatment guidelines were published, including the Canadian Association of Gastroenterology Toronto consensus published in 2016.^4–6^ Important recommendations were made, including:

1. that most treatments should have a duration of 14 days,
2. that a clarithromycin containing regimen should not be used, if a prior clarithromycin-containing regimen had failed,
3. recommended first-line therapies are either concomitant (PAMC) therapy or bismuth-based quadruple therapy (PBMT)
4. bismuth-based quadruple therapy or alternatively the combination of PPI, amoxicillin, and levofloxacin are good second and third-line options,
5. fourth line therapy is the combination of PPI, amoxicillin, and rifabutin.
6. Use of the triple-combination of a PPI with clarithromycin and either amoxicillin or metronidazole is no longer recommended.

Generally, Concomitant (PAMC) therapy is preferred over Bismuth-based quadruple therapy as first-line treatment, as PBMT is more cumbersome to take and has more side effects. PBMT is recommended as first line in patients with penicillin allergy.

Concomitant (PAMC) performed well as a first-line therapy with a success rate of 87% compared to a 83% first-line cure rate with sequential therapy during 2007–2015, although this difference was not statistically significant. A meta-analysis comparing concomitant to sequential therapy in 14 studies showed concomitant therapy was significantly more effective than sequential therapy ITT eradication rate, 85.7% versus 79.7% with a statistically significant RD of 6%).^4^

The data confirm (figure 2) that once a patient fails a first line therapy that success rates with subsequent therapies are not as high. The most common reason is resistance of *H. pylori* to antibiotics.^3,4^ Logistic regression of predictors of success (Table 3) did show that previous exposure to clarithromycin, metronidazole, or amoxicillin was negatively associated with treatment success. Furthermore, concomitant therapy, used as first-line therapy, is also a predictor of success, reiterating that it is important that treatment guidelines are followed.

Other 2016 data from Canada reported a cure rate of 81% per protocol (PP) and 75% ITT in 175 patients, who failed first-line treatments.^13^ Using seven different treatment combinations bismuth quadruple therapy given for 7,10, or 14 days for the first failure or for 14 days for second to fifth failure was superior to all PPI triple therapies: BMT 91%, PP < 84% ITT, triple therapy PP 66%, ITT 62%.^13^

Data from the European Registry on *H. pylori* Management (Hp-EuReg) consortium supports the use of the management algorithm PAMC—PBMT—PAL—PAR.^14^ The main explanation for the decrease in efficacy of PAC and PMC is the markedly increased resistance of *H. pylori* against clarithromycin, also observed in Canada.^2,7,8^ Fallone et al., in a 2000 review, reported resistance rates of 4% to clarithromycin and 18–22% to metronidazole.^15^ Resistance data obtained from arctic communities living in the Yukon and the Northwest Territories show similar rates.^16^ Our culture data, although based on a small sample size, show that background resistance to clarithromycin is currently much higher, justifying why the PAC or PMC combinations should be avoided. However, these data should be interpreted with caution as they mainly reflect patients who were treated with this therapy and failed and subsequently were referred to the UAH.

As can be seen in Table 4, during 1999–2006, primary resistance to clarithromycin was substantial at 15% (37/ 245) and secondary resistance at 56% (137/245). Data for the period 2007–2021 show very high levels of resistance to clarithromycin, ranging from 59% to 85%. Data on resistance to levofloxacin also show high rates of resistance of 19% (30/157) during 1999–2006 and ranging from 20% to 59% during 2007–2021. This is likely due to the frequent use of quinolones for other indications. The high background resistance rate to levofloxacin is the reason why the use of levofloxacin is not recommended as part of first-line therapy.^3^

Worldwide resistance to amoxicillin is low, and we found the same in our patients. However, in the four strains with amoxicillin resistance, the MICs were all slightly above the EUCAST breakpoint of 0.125 mg/L. It is unclear whether this signifies clinically significant amoxicillin resistance. Resistance to tetracycline was not observed in any of the isolated strains during the 2007–2021 period. Thus, both amoxicillin and tetracycline are excellent antibiotic choices for *H. pylori* therapy.

Over the last 25 years, rates of metronidazole resistance rates have consistently remained high but have risen from 25% primary resistance during the period 1999–2006 to 40–88% during the periods 2007–2015 and 2016–2021. The reason why metronidazole continues to be used, despite high rates of resistance, is the evidence that resistance to metronidazole can be partially overcome when it is used in combination with other agents.^17–19^ That means that even though a strain is resistant to metronidazole, the success rate is higher by leaving metronidazole in, but not as high when compared with strains that were sensitive to metronidazole.^17–19^ We found that dual resistance to metronidazole was found in 46% (23/50) of clarithromycin-resistant strains. It is known that the presence of this type of dual resistance is a strong predictor of treatment failure when clarithromycin-metronidazole regimes are used.^3^

Data on antibiotic resistance in the USA are also scarce. Rising USA resistance rates in a recent meta-analysis were reported: clarithromycin 25%, metronidazole 45%, levofloxacin 22%, tetracycline < 2%, and amoxicillin <2%.^20^ In a recent study of 907 mainly American and European patients, resistance rates to clarithromycin were 22.2%, metronidazole 69.2%, and amoxicillin 1.2%, and among clarithromycin-resistant isolates, 75% were also metronidazole-resistant.^21^ Worldwide data also report rising rates of resistance of *H. pylori* strains.^22^

It is important for clinicians to keep these resistance data in mind when choosing *H. pylori* therapies. Ideally, local data on resistance profiles of *H. pylori* are known, but in reality, this is seldom the case. In the absence of local resistance data, the best substitute is regular assessment of success rates of treated patients to document whether they conform with expected levels of success.

Although side effects were not formally assessed in detail in our patients, treatment generally was well tolerated. The large majority of patients were able to finish the entire course of therapy. High compliance may be in part facilitated by the fact that all our patients receive a detailed handout explaining the importance of *H. pylori* infection and providing instructions on when the different treatments should be taken. Furthermore, all our prescriptions are written as blister packs to facilitate compliance. Hp-EuReg documented that compliance generally is high, >97% and serious side-effects are rare, 0.08%.^23^ In some countries, including the USA and parts of Europe, a triple combination tablet is available for the combination of bismuth, metronidazole, and tetracycline, and the efficacy of this medication has been reported as 90% as first line and as rescue therapy.^24,25^

Pantoprazole at 83% was by far the most commonly used PPI as part of *H. pylori* treatment. It is also the most common PPI prescribed in Alberta as it is preferentially listed by the Alberta provincial drug plan. However, using a comparison to 20 mg omeprazole equivalents, pantoprazole 40 mg is one of the weaker PPIs.^26^ Hp-EuReg data show that by using more potent PPIs (such as esomeprazole 40 mg bid or higher), there is a slightly higher cure rate.^27^ More potent acid suppression may also be the explanation why the potassium competitive inhibitor inhibitor vonoprazan (not available in Canada currently) has higher cure rates of *H. pylori* infection than lansoprazole when combined with clarithromycin and amoxicillin.^28^

In Canada, the combination of PPI, amoxicillin, and rifabutin has been mainly used as fourth-line rescue therapy. The success of this combination in 2010 used as fourth-line rescue therapy was 62%.^29^ Since 2019, a combination tablet of omeprazole, amoxicillin, and rifabutin (Talicia^®^) has become available in the USA, and the success rate of this 10-day regimen given as first line was 84%.^30^ In Canada, to date, rifabutin is a restricted drug, and special authorization for its use needs to be requested, and the combination table is not available. Another limitation is that rifabutin is expensive. The limited data on rifabutin resistance to date have shown very low levels of resistance.^20,30^ Rifabutin can cause myelotoxicity, but in treatment for *H. pylori*, this is rare,^7,31,32^ and no cases were observed in our patients treated with rifabutin.

Given the high levels of resistance to levofloxacin we observed, consideration should be given to using PAR as third-line therapy. It is acknowledged that the success rate of PAR therapy of 50–57% observed is suboptimal, even when given as rescue therapy. More studies of rifabutin are required to see if changes to the duration of treatment, dosages of drugs used, or use in combination with other agents, such as bismuth compounds, could increase treatment efficacy. Given the high levels of metronidazole resistance, a change in bismuth quadruple therapy could also be considered by using amoxicillin instead of metronidazole. Finally, another management option after multiple treatment failures is the decision not to treat *H. pylori* any further but possibly observe these patients long term. Long-term follow-up especially is a consideration if there is a family history of gastric cancer or if gastric metaplasia was present on histology.^33^

Limitations to the study include the fact that this was a retrospective single-centre study. Therefore, the results may not be generalizable. Patients were only included if a TOC was available after treatment. Data capture on levels of adherence, tolerability, and adverse events associated with regimens was limited. Some patients were treated in the community prior to referral, so their treatment information could have been incomplete, but all efforts were made to identify previous anti-*Helicobacter* treatment regimens. The number of Edmonton patients in whom culture and antibiotic sensitivity data was available was small, and this was especially true for treatment-naïve individuals. However, data from Canada on *H. pylori* cultures and resistance are very limited. For this reason, resistance data from 1996 to 2006 from Halifax, Nova Scotia were included as it is a large dataset, even though it was not derived from the patient cohort reported in this study. The Halifax data help to show the changes in antibiotic resistance of *H. pylori* strains over a longer period in Canada.

The Edmonton culture data from 2007 to 2021, although based on a small sample size, show important findings of the background resistance to antibiotics, that is, high levels of resistance to clarithromycin, metronidazole, and levofloxacin and low levels to amoxicillin and tetracycline.

## Conclusions

This real-world study shows that following existing guidelines with algorithmic treatment with PAMC first line followed by PBMT, PAL, and PAR is effective at eradicating *H. pylori* infection in 88% of newly diagnosed patients. Antibiotic resistance in *H. pylori* is common to clarithromycin, metronidazole, and levofloxacin and frequently accounts for treatment failures. Ideally, culture and sensitivity testing should be done to understand resistance patterns in *H. pylori* but TOC data is an important substitute to document the success rates of treatments that are used in the community. Rifabutin-based regimens perform suboptimal (50–57% cure rate) and require more study for optimization, however, may be considered as third-line therapy due to increasing levels of levofloxacin resistance.

## Supplementary data

Supplementary data are available at *Journal of the Canadian Association of Gastroenterology* online.

gwad051_suppl_Supplementary_Materials

## Data Availability

Data are available on request from the authors.
