# Supplementary material for: Success of Helicobacter pylori Guideline-based Treatment of Newly Diagnosed and Previously Treated Patients During 2007–2021 in Edmonton, Alberta
Source: J Can Assoc Gastroenterol. 2023 Dec 14;7(3):221–9. doi: 10.1093/jcag/gwad051 (PMC11149661; doi:10.1093/jcag/gwad051)
Supplement: gwad051_suppl_Supplementary_Materials [file gwad051_suppl_supplementary_materials.zip › gwad051_AQ14_Hp Suppl_updated.docx]

Supplementary file

Cultures of *Helicobacter pylori*

Cultures obtained from patients were assessed for antibiotic resistance to clarithromycin, metronidazole, levofloxacin, tetracycline, and amoxicillin, using the most recent breakpoints for the European Committee on Antimicrobial Susceptibility Testing (EUCAST-2023: https://www.eucast.org/clinical_breakpoints) by epsilometer test (E test, AB Biodisk, Sweden, now bioMerieux., Marcy-l'Étoile, France). Strains were grown on chocolated Brain Heart Infusion (BHI) agar without antimicrobial agents for two subcultures using sheep blood more than two weeks old. Each *H. pylori* strain was suspended at a density equivalent to a MacFarlands #2 in sterile saline. For E testing, each agar plate was spread with the MacFarlands #2 suspension and an E test strip was added to the plate. The plates were incubated under microaerobic conditions using an anaerobic incubator set to 5% oxygen, 10% carbon dioxide and 85% nitrogen at 35°C. Minimum inhibitory concentrations (MICs) were recorded at 72 h. The E test MIC was defined as the point at which the growth intersected the strip. Metronidazole resistance was defined by growth greater than 8 mg/L, Clarithromycin, Levofloxacin and Tetracycline resistance were defined by growth greater than 1 mg/L and Amoxicillin resistance was defined by growth greater than 0.125 mg/l. A haze of growth that could not be distinguished from the inoculum was discounted.

**Supplementary Table 1: Cultures of *Helicobacter pylori* resistant to Clarithromycin and Metronidazole**

| **Dual resistance cases (CLA-R, MET-R)** | **Number of treatments given before culture** | **Regimen 1** | **Regimen 2** |
| --- | --- | --- | --- |
| 4 | 4 | No further treated |  |
| 7 | 2 | PBMT (success) |  |
| 15 | 5 | PAR (success) |  |
| 18 | 2 | PAR (fail) | PBAT (fail) |
| 82 | 3 | PAR (fail) | PBAT (fail) |
| 86 | 2 | PBMT (success) |  |
| 92 | 3 | PBAT (success) |  |
| 93 | 3 | PAR (success) |  |
| 94 | 3 | PAR (fail) |  |
| 100 | 4 | No further treatment |  |
| 102 | 3 | PAR (fail) |  |
| 108(x2) | 4 | PBAT (fail) |  |
| 109 | 3 | PBAT (fail) |  |
| 111 | 2 | PAL (fail) | PBMT (fail) |
| 132 | 3 | PAL (success) |  |
| 137 | 0 | PPI-Ampicillin-CLA (fail) | PPI-Ampicillin-TET (fail) |
| 138 | 2 | None |  |
| 246 | 3 | PALT (fail) |  |
| 248 | 3 | PAL (success) |  |
| 249 | 2 | PAL (fail) |  |
| 272 | 2 | No further treatment |  |
| 290 | 2 | PAR (fail) |  |

Acronyms used: PPI, proton-pump inhibitor; CLA: clarithromycin; TET: tetracycline; CLA-R, clarithromycin resistance; MET-R, metronidazole resistance; PAR, PPI-amoxicillin-rifabutin; PMC, PPI-metronidazole-clarithromycin; PAL, PPI-amoxicillin-rifabutin; PBMT, PPI-bismuth-amoxicillin-tetracycline; PBAT, PPI-bismuth-amoxicillin-tetracycline; PALT, PPI-amoxicillin-levofloxacin-tetracycline
